# SURVIVAL AND PROGNOSTIC FACTORS OF ANAL CANCER: A STUDY BASED ON DATA FROM THE HOSPITAL-BASED CANCER REGISTRY OF A HIGH-COMPLEXITY ONCOLOGY CARE CENTER

**DOI:** 10.1590/0102-6720202400037e1830

**Published:** 2024-10-28

**Authors:** Wallace Henrique Pinho da PAIXÃO, Gelcio Luiz Quintella MENDES, Débora Santos da SILVA, Rosyane Garcês Moreira Lima de SOUZA, Rodrigo Otavio de Castro ARAUJO, Karina Cardoso MEIRA, Rafael Tavares JOMAR

**Affiliations:** 1 Instituto Nacional de Câncer – Rio de Janeiro (RJ), Brazil.; 2 Universidade Federal do Rio Grande do Norte – Natal (RN), Brazil.

**Keywords:** Anus Neoplasms, Carcinoma, Squamous Cell, Survival Analysis, Prognosis, Chemoradiotherapy, Neoplasias do Ânus, Carcinoma de Células Escamosas, Análise de Sobrevida, Prognóstico, Quimiorradioterapia.

## Abstract

**BACKGROUND:**

Anal cancer is a relatively rare disease, and there is a lack of survival data from low- and middle-income countries.

**AIMS:**

The aim of this study was to investigate the survival rates and prognostic factors of anal cancer cases treated at a High-Complexity Oncology Care Center in Rio de Janeiro, Brazil.

**METHODS:**

A retrospective cohort study was conducted involving 665 cases of squamous cell carcinoma of the anus/anal canal treated from 2000 to 2016. To estimate the 5-year overall survival probability and survival according to selected variables, the Kaplan-Meier method and the log-rank test were applied. To identify factors associated with survival, the Cox proportional hazards model, stratified by staging, was used to estimate hazard ratios (HR). Ninety-five percent confidence intervals (95%CI) were also calculated.

**RESULTS:**

The overall survival probability was 62.20% (95%CI 57.90–66.20). Higher survival rates were observed in female cases, those with non-advanced staging, and those treated with chemoradiotherapy (p<0.001). Among cases with advanced staging, being female was a protective factor against death (HR=0.52; 95%CI 0.28–0.93). Compared to chemoradiotherapy, at least one type of treatment was identified as a risk factor: chemoradiotherapy + surgery among cases with non-advanced staging (HR=22.65; 95%CI 5.65–90.81), radiotherapy among cases with advanced staging (HR=2.71; 95%CI 1.39–5.30), and among cases with unknown staging, no treatment (HR=3.36; 95%CI 1.73–6.50), radiotherapy (HR=2.38; 95%CI 1.46–3.88), and radiotherapy + surgery (HR=3.99; 95%CI 1.20–13.27).

**CONCLUSIONS:**

The findings support the superiority of chemoradiotherapy over other therapeutic modalities for anal cancer, resulting in increased survival and a better prognosis.

## RESUMO

**RACIONAL:**

O câncer anal é uma doença relativamente rara com escassas evidências de sobrevida oriundas de países de baixa e média renda.

**OBJETIVOS:**

Investigar a sobrevida e os fatores prognósticos de casos de câncer anal atendidos em Centro de Assistência de Alta Complexidade em Oncologia na capital do Rio de Janeiro, Brasil.

**MÉTODOS:**

Estudo de coorte retrospectiva desenvolvido com 665 casos de carcinoma escamocelular do ânus/canal anal atendidos no período 2000-2016. Para estimar a probabilidade de sobrevida em cinco anos, global e segundo variáveis selecionadas, aplicaram-se o método de Kaplan-Meier e o teste log-rank. Para identificar fatores associados à sobrevida, o modelo semiparamétrico de riscos proporcionais de Cox, estratificado por estadiamento, estimou *hazard ratios* (HR). Intervalos de 95% de confiança (IC95%) também foram calculados.

**RESULTADOS:**

A probabilidade de sobrevida global foi de 62,20% (IC95% 57,90–66,20). Maior sobrevida foi observada em casos do sexo feminino, com estadiamento não avançado e naqueles tratados com quimiorradioterapia (p<0,001). Entre os casos com estadiamento avançado, o sexo feminino mostrou-se fator de proteção para o óbito (HR=0,52; IC95% 0,28–0,93). Em comparação à quimiorradioterapia, pelo menos um tipo de tratamento mostrou-se fator de risco: quimiorradioterapia+cirurgia entre os casos com estadiamento não avançado (HR=22,65; IC95% 5,65–90,81), radioterapia entre os casos com estadiamento avançado (HR=2,71; IC95% 1,39–5,30) e, entre os casos com estadiamento desconhecido, nenhum (HR=3,36; IC95% 1,73–6,50), radioterapia (HR=2,38; IC95% 1,46–3,88) e radioterapia+cirurgia (HR=3,99; IC95% 1,20–13,27).

**CONCLUSÕES:**

Os achados corroboram a superioridade da quimiorradioterapia sobre as demais modalidades terapêuticas contra o câncer anal, determinando maior sobrevida e melhor prognóstico.

## INTRODUCTION

Anal cancer occurs in the canal and outer edges of the anus and is a relatively rare disease, with global estimates of 50,865 new cases (0.3% of all cancer cases) and 19,293 deaths from this cause (0.2% of all cancer deaths) in 2020^43^. Although there are no incidence estimates for Brazil^37^, recent data revealed 1,101 deaths from anal cancer (598 female) in the country in 2021^16^.

Chemoradiotherapy, a combination of radiation therapy and chemotherapy (5-fluorouracil and mitomycin C), is the standard treatment for localized or locally advanced anal cancer, with 5-year survival rates ranging from 72% to 89%. Chemotherapy alone is usually used to treat metastatic disease, while surgery is employed for recurrent or residual disease^13,42,44^.

In adults diagnosed with anal cancer in England between 2015 and 2019, the observed survival was 85% at 1 year and 62% at 5 years^28^. For those diagnosed in the United States between 2013 and 2019, the 5-year relative survival rate was 70%^27^. In the European Cancer Registry-Based Study on Survival and Care of Cancer, a large study on cancer survival that compiles data from cancer registries across various European countries, the observed survival rates for 5,386 adults diagnosed with anal cancer from 1983 to 1994 were 76% at 1 year and 43% at 5 years, with a small but significant difference between women (55%; 95%CI 53–57) and men (49%; 95%CI 47–52)^12^.

Most survival studies on anal cancer are concentrated in the United States and Europe, with scarce evidence from low- and middle-income countries like Brazil. Among the few Brazilian studies on the subject, noteworthy are those by Ferrigno et al.^11^, Parra et al.,^32^ and Libera et al.^24^. Ferrigno et al. evaluated 43 patients treated with chemoradiotherapy between 1993 and 2001, finding an overall survival rate of 68% at 5 years, with rates of 100% in stage I, 82% in stage II, 73% in stage IIIA, and 18% in stage IIIB^11^. Parra et al. followed 50 patients treated at a university hospital from 1979 to 2004, reporting an overall 5-year survival rate of 18% for those with distant metastases and 78% for those with localized disease^32^. Libera et al. evaluated 81 patients treated at an oncology reference center from 2000 to 2010, describing an overall 5-year survival rate of 44%^24^.

This study was designed to contribute to expanding knowledge about hospital survival of anal cancer in Brazil, a country with a universal and free healthcare system, the Unified Health System (SUS in Portuguese), on which seven out of ten Brazilians are entirely dependent^41^. Therefore, its objective was to investigate the survival rates and prognostic factors of anal cancer cases treated at a High-Complexity Oncology Care Center that is part of the SUS and a reference for cancer treatment in the state of Rio de Janeiro.

## METHODS

A retrospective hospital-based cohort study was conducted with anal cancer cases treated between January 1, 2000, and December 31, 2016, at a High-Complexity Oncology Care Center located in the capital city of the state of Rio de Janeiro, Brazil. The data source was the Hospital-Based Cancer Registry of this Center.

All cases with a histopathological diagnosis of squamous cell carcinoma (histological code 8070/3 of the International Classification of Diseases for Oncology^46^]) of the anus or anal canal (topographical codes C21.0 and C21.1 of the 10th Revision of the International Classification of Diseases^45^) were included in the study, while those without a diagnosis date were excluded. The decision to include only cases of squamous cell carcinoma was based on the fact that the majority of anal cancer cases are of this histological type^13,23,33,36,44^.

The active 5-year follow-up began on the date of the squamous cell carcinoma diagnosis of the anus or anal canal, using the following procedures as of December 2021: consultation of the database provided by the Hospital-Based Cancer Registry, consultation of the Mortality Information System of the State of Rio de Janeiro, and consultation of the Extrajudicial Portal of Birth and Death Records of the Judiciary of the State of Rio de Janeiro.

The variables investigated at the time of diagnosis, and if applicable, subsequently transformed for statistical analysis, were: sex (male and female), age (in age groups: ≤49, 50–69, and ≥70 years), race/skin color (white and non-white), education (none/incomplete elementary, complete elementary, and high school or higher), marital status (single, widowed/divorced, and married/consensual union), municipality of origin (Rio de Janeiro and others), smoking history (no and yes), alcohol consumption history (no and yes), referral source (SUS, non-SUS, and none), year of diagnosis (in 6-year periods: 1999–2004, 2005–2010, and 2011–2016), and staging (I, II, III, IV, and unknown). Additionally, the time between diagnosis and treatment (in days: ≤60 and >60) and treatment received (none, surgery, radiotherapy, chemotherapy, and combinations of these) were also investigated.

For the description of variables, mean, standard deviation, median, interquartile range, and absolute and relative frequencies were calculated. Chi-square or Fisher’s exact tests were used, considering a p<0.05, to compare the proportions of categorical variables between staging groups (known vs. unknown).

To estimate the 5-year survival probability and the corresponding 95% confidence intervals (95%CIs), the Kaplan-Meier method was applied using the following criteria:

1. Initial event: diagnosis of squamous cell carcinoma of the anus or anal canal;

2. Final event: death, regardless of cause;

3. Survival time: the time elapsed between the initial and final events; and

4. Censoring: cases lost to follow-up or still alive at the end of the follow-up period.

Subsequently, overall survival functions were estimated according to the variables investigated and their respective 95%CI. The log-rank test was used to compare survival curves.

To identify factors associated with 5-year survival, the Cox proportional hazards semi-parametric model was used to estimate crude hazard ratios (HRs) and their respective 95%CI for each variable that had a p<0.20 in the log-rank test. Next, variables that did not violate the proportional hazards assumption, assessed using the proportional hazards assumption test (p>0.05), were included in the multivariate Cox model, stratified by staging (non-advanced [I and II], advanced [III and IV], and unknown), which estimated adjusted HRs and their respective 95%CI. Grouping cases into advanced and non-advanced staging was intended to provide greater stability to the model results. All statistical analyses were conducted using Stata 15.0.

The study was approved on February 12, 2021, by the Research Ethics Committee of the Brazilian National Cancer Institute, which waived the requirement for obtaining informed consent (Number 4.538.738; CAAE: 37224720.0.0000.5274).

## RESULTS

A total of 665 cases (663 anal canal and two anus) met the study’s eligibility criteria, with a mean age of 60.69 years (standard deviation=12.32) and a median age of 60 years (interquartile range=18). The minimum age was 25, and the maximum age was 98. As shown in Table 1, the majority of cases were female (84.36%), of white race/skin color (67.07%), had no education or only incomplete elementary education (53.08%), were from the municipality of Rio de Janeiro (50.98%), had no history of smoking (49.92%), and had no history of alcohol consumption (63.91%). Among those who received treatment, 77.91% waited more than 60 days to receive it, with chemoradiotherapy being the most common treatment modality (68.27%). The absence of information for the variables referral source and staging was exactly 37.44% and 49.32%, respectively.

**Table 1:** Characteristics of anal cancer cases (n=665).

Variables	n	%
Sex		
Male	104	15.64
Female	561	84.36
Age group (years)		
≤49	123	18.50
50–59	203	30.53
60–69	170	25.56
≥70	169	25.41
Race/skin color		
White	446	67.07
Non-white*	213	32.03
No information	06	0.90
Education level		
None/incomplete elementary	353	53.08
Complete elementary	96	14.44
High school or higher	206	30.98
No information	10	1.50
Marital status		
Single	191	28.72
Married/consensual union	238	35.79
Widowed/divorced	222	33.38
No information	14	2.11
Municipality of origin		
Rio de Janeiro	339	50.98
Others	321	48.27
No information	05	0.75
Smoking history		
No	332	49.92
Yes^†^	310	46.62
No information	23	3.46
Alcohol consumption history		
No	425	63.91
Yes^‡^	211	31.73
No information	29	4.36
Referral source		
SUS	257	38.65
Non-SUS	146	21.95
None	13	1.95
No information	249	37.44
Diagnosis period (6-year periods)		
1999–2004	211	31.73
2005–2010	247	37.14
2011–2016	207	31.13
Staging		
I	10	1.50
II	135	20.30
III	162	24.36
IV	30	4.51
No information	328	49.32
Time between diagnosis and treatment^§^		
≤60 days	131	22.09
60 days	462	77.91
Treatment		
None	67	10.08
Surgery	16	2.41
Radiotherapy	90	13.53
Chemotherapy	07	1.05
Chemoradiotherapy	454	68.27
Radiotherapy+surgery	04	0.60
Chemotherapy+surgery	01	0.15
Chemoradiotherapy+surgery	26	3.91

*Category includes Black (n=60), Brown (n=150), and Asian (n=03);

^†^Category includes smokers (n=250) and former smokers (n=60);

^‡^Category includes current drinkers (n=178) and former drinkers (n=33);

^§^Excludes untreated cases (n=67) or those without a treatment date (n=05). SUS: *Sistema Único de Saúde*.

Regarding the frequency of staging status presented in Table 2, only the variables diagnostic period (p<0.001), treatment received (p<0.001), and time between diagnosis and treatment (p=0.008) showed differences between the proportions of their categories.

**Table 2:** Characterization of anal cancer cases according to staging status (n=665).

Variables	Staging		p-value
	Known (n=337) n (%)	Unknown (n=328) n (%)	
Sex			
Male	53 (15.73)	51 (15.55)	0.950*
Female	284 (84.27)	277 (84.45)	
Age group (years)			
≤49	69 (20.47)	54 (16.46)	0.066*
50–59	111 (32.94)	92 (28.05)	
60–69	85 (25.22)	85 (25.91)	
≥70	72 (21.36)	97 (29.57)	
Race/skin color			
White	230 (69.07)	216 (66.26)	0.440*
Non-white	103 (30.93)	110 (33.74)	
Education level			
None/incomplete elementary	185 (55.72)	168 (52.01)	0.543*
Complete elementary	49 (14.76)	47 (14.55)	
High school or higher	98 (29.52)	108 (33.44)	
Marital status			
Single	102 (31.10)	89 (27.55)	0.467*
Married/consensual union	113 (34.45)	125 (38.70)	
Widowed/divorced	113 (34.45)	109 (33.75)	
Municipality of origin			
Rio de Janeiro	173 (51.49)	166 (51.23)	0.948*
Others	163 (48.51)	158 (48.77)	
Smoking history			
No	162 (49.09)	170 (54.49)	0.171*
Yes	168 (50.91)	142 (45.51)	
Alcohol consumption history			
No	216 (65.65)	209 (68.08)	0.516*
Yes	113 (34.35)	98 (31.92)	
Referral source			
SUS	133 (60.45)	124 (63.27)	0.397*
Non-SUS	82 (37.27)	64 (32.65)	
None	05 (2.27)	08 (4.08)	
Diagnosis period (6-year periods)			
1999–2004	136 (40.36)	75 (22.87)	<0.001*
2005–2010	106 (31.45)	141 (42.99)	
2011–2016	95 (28.19)	112 (35.15)	
Time between diagnosis and treatment (days)			
≤60	84 (26.25)	47 (17.22)	0.008*
60	236 (73.75)	226 (82.78)	
Treatment			
None	14 (4.15)	53 (16.16)	<0.001^†^
Surgery	03 (0.89)	13 (3.96)	
Radiotherapy	43 (12.76)	47 (14.33)	
Chemotherapy	04 (1.19)	03 (0.91)	
Chemoradiotherapy	262 (77.74)	192 (58.54)	
Radiotherapy+surgery	-	04 (1.22)	
Chemotherapy+surgery	-	01 (0.30)	
Chemoradiotherapy+surgery	11 (3.26)	15 (4.57)	

*χ^2^ test;

^†^Fisher’s exact test; SUS: *Sistema Único de Saúde.*

During the 5-year follow-up, there were 350 (52.63%) censored cases and 315 (47.37%) deaths. Of those who died, 79.05% were female, 30.79% were aged ≥70 years, 67.30% were white, 55.84% had no education or only incomplete elementary education, 35.83% were widowed or divorced, 52.24% were from the municipality of Rio de Janeiro, 51.67% had no history of smoking, 67.89% had no history of alcohol consumption, 61.00% were referred by SUS, 40.32% were diagnosed between 2005 and 2010, 51.75% had unknown staging, 77.20% received treatment more than 60 days after diagnosis, and 50.48% were treated with chemoradiotherapy.

The censored group consisted of 264 (75.43%) cases alive at the end of follow-up and 86 (24.57%) lost to follow-up. This group had a mean age of 57.98 years (SD (standard deviation)=10.35) and a median age of 58.5 years (IR (interquartile range)=14), with mean and median follow-up times of 3.38 years (SD=1.77) and 4 years (interquartile range=3), respectively. Compared to the other cases in the cohort, those lost to follow-up showed differences in the proportions of the categories for the variables age (p=0.027), education (p=0.011), year of diagnosis (p<0.001), staging (p=0.012), and treatment received (p<0.004).

The mean and median follow-up times for all cases in the cohort were 3.02 years (SD=2.05) and 4 years (IR=4), respectively. For censored cases, the mean and median follow-up times were 4.60 years (SD=1.11) and 5 years (IR=0), respectively. For cases resulting in death, the mean and median follow-up times were 1.27 years (SD=1.31) and 1 year (IR=2), respectively.

**Figure 1:**
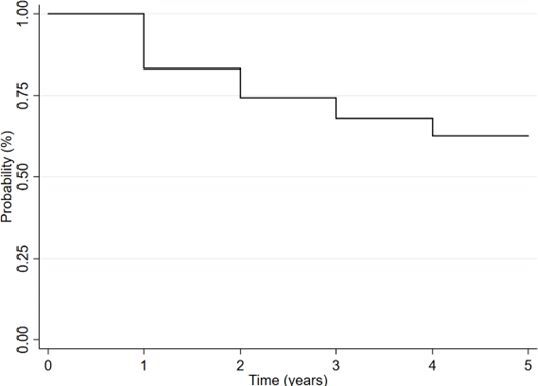
Kaplan-Meier curve showing the 5-year survival of anal cancer cases.

The 5-year overall survival probability was 62.20% (95%CI 57.90–66.20), as shown in Figure 1. Table 3 displays the conditional survival probability according to the variables investigated. Higher survival curves were observed in female cases, those with non-advanced staging, and those treated with chemoradiotherapy (p<0.001).

**Table 3:** Five-year survival probability of anal cancer cases.

Variables	% (95%CI)	p-value*
Global	62.20 (57.90–66.20)	
Sex		
Male	45.85 (34.14–56.78)	<0.001
Female	64.84 (60.25–69.05)	
Age group (years)		
≤49	63.70 (53.24–72.41)	0.458
50–59	64.45 (56.77–71.12)	
60–69	64.85 (56.14–72.26)	
≥70	55.22 (46.06–63.44)	
Race/skin color		
White	61.77 (55.92–66.00)	0.791
Non-white	63.35 (55.43–70.24)	
Education level		
None/incomplete elementary	61.63 (55.63–67.07)	0.593
Complete elementary	68.05 (55.90–77.51)	
High school or higher	61.83 (54.05–68.68)	
Marital status		
Single	65.70 (57.36–72.80)	0.758
Married/consensual union	62.32 (55.12–68.70)	
Widowed/divorced	60.46 (52.77–67.30)	
Smoking history		
No	63.27 (57.13–68.78)	0.601
Yes	61.40 (55.05–67.13)	
Alcohol consumption history		
No	62.30 (56.87–67.24)	0.894
Yes	62.20 (54.45–69.00)	
Referral source		
SUS	61.65 (54.60–67.94)	0.856
Non-SUS	59.59 (50.16–67.80)	
None	50.00 (15.20–77.49)	
Diagnosis period (6-year periods)		
1999–2004	65.05 (57.40–71.68)	0.285
2005–2010	57.73 (50.54–64.26)	
2011–2016	65.08 (57.10–71.94)	
Staging		
Non-advanced	75.64 (67.18–82.21)	<0.001
Advanced	53.01 (44.60–60.72)	
Unknown	60.70 (54.38–66.43)	
Time between diagnosis and treatment (days)		
≤60	64.25 (54.17–72.67)	0.285
60	63.56 (58.64–68.06)	
Treatment^†^		
None	22.22 (6.91–42.88)	<0.001
Surgery	61.54 (30.83–81.84)	
Radiotherapy	40.97 (29.06–52.51)	
Chemotherapy	26.67 (0.97–68.61)	
Chemoradiotherapy	69.21 (64.41–73.49)	
Chemoradiotherapy+surgery	44.20 (23.74–62.90)	

*Log-rank test;

^†^It was not possible to calculate the survival probability for the missing categories due to their small sample size. SUS: *Sistema Único de Saúde*; CI: confidence interval.

The HRs estimated by the Cox proportional hazards models are presented in Table 4. The multivariate model showed that cases with unknown staging who received no treatment or radiotherapy followed by surgery had a 3.36 times (95%CI 1.73–6.50) and 3.99 times (95%CI 1.20–13.27) higher risk of death, respectively, compared to those treated with chemoradiotherapy. Conversely, female cases with advanced staging had a 48% lower risk of death compared to male cases (95%CI 0.28–0.93). Compared to those treated with chemoradiotherapy, a higher risk of death was observed in cases treated with radiotherapy alone, where the staging was advanced (HR=2.71; 95%CI 1.39–5.30) or unknown (HR=2.38; 95%CI 1.46–3.88), and in cases with non-advanced staging that received chemoradiotherapy followed by surgery (HR=22.65; 95%CI 5.65–90.81).

**Table 4:** Crude (hazard ratio crude) and adjusted (hazard ratio adjusted) hazard ratios for 5-year mortality of anal cancer cases according to staging status.

Variables	Staging					
	Non-advanced (n=145)		Advanced (n=192)		Unknown (n=328)	
	HRc (95%CI)	HRa (95%CI)	HRc (95%CI)	HRa (95%CI)	HRc (95%CI)	HRa (95%CI)
Sex						
Male	1.00	1.00	1.00	1.00	1.00	1.00
Female	0.71 (0.27–1.86)	0.56 (0.20–1.52)	0.51 (0.28–0.91)	0.52 (0.28–0.93)	0.55 (0.34–0.91)	0.72 (0.43–1.22)
Treatment						
Chemoradiotherapy	1.00	1.00	1.00	1.00	1.00	1.00
None	4.41 (0.59–32.77)	4.88 (0.65–36.69)	5.66 (0.76–41.77)	6.42 (0.86–47.67)	3.74 (1.98–7.04)	3.36 (1.73–6.50)
Surgery	*	*	5.66 (0.76–41.77)	6.42 (0.86–47.67)	1.31 (0.47–3.62)	1.22 (0.44–3.42)
Radiotherapy	1.45 (0.50–4.22)	1.58 (0.53–4.68)	2.90 (1.49–5.63)	2.71 (1.39–5.30)	2.44 (1.50–3.97)	2.38 (1.46–3.88)
Chemotherapy	6.22 (0.82–46.76)	6.90 (0.90–52.53)	2.87 (0.69–11.88)	3.20 (0.77–13.31)	*	*
Radiotherapy+surgery	*	*	*	*	4.60 (1.42–14.90)	3.99 (1.20–13.27)
Chemoradiotherapy+surgery	20.28 (5.17–79.59)	22.65 (5.65–90.81)	1.53 (0.55–4.26)	1.45 (0.52–4.02)	1.59 (0.68–3.70)	1.56 (0.67–3.65)

*Hazard ratios could not be estimated due to the small sample size. HRc: hazard ratio crude; HRa: hazard ratio adjusted; CI: confidence interval.

## DISCUSSION

The study results show that 5 years after the diagnosis of squamous cell carcinoma of the anus or anal canal the overall survival probability is around 60%, and nearly 70% for cases treated with chemoradiotherapy. Additionally, they indicate that among cases with advanced staging, being female is a protective factor against death, and in any staging group, at least one type of treatment poses a risk factor compared to chemoradiotherapy.

Since the mid-1970s, the standard treatment for localized anal cancer has been chemoradiotherapy^29,30,35^, as it results in a cure in most cases^1^. Recently, the Brazilian Society of Surgical Oncology reaffirmed that in cases of persistent disease (a residual tumor identified within 6 months of completing chemoradiotherapy) or recurrence (a viable tumor diagnosed 6 months after completing chemoradiotherapy), salvage surgery with curative intent is necessary, typically involving abdominoperineal resection^44^. The persistence or recurrence of the disease is likely the reason for the 22-fold higher risk of death observed among cases with non-advanced staging who underwent chemoradiotherapy followed by surgery compared to those who received standard treatment. It is worth noting that, although the 95%CI for this association measure was wide, its lower limit was still quite high, confirming a poor prognosis for these cases^31^.

Excluding cases with non-advanced staging, radiotherapy alone or followed by surgery was consistently associated with a higher risk of death. This suggests that such cases did not have favorable clinical conditions for standard treatment due to the toxic effects of chemotherapy^20^, resulting in a two- to four-times higher risk of death compared to those treated with chemoradiotherapy.

Just over 10% of the cases were not subjected to any treatment, consequently showing the lowest survival probability observed in the study. Nearly 80% of these had unknown staging, and they exhibited a 3.36 times higher risk of death compared to those who underwent chemoradiotherapy. It is possible to argue that these were cases of advanced disease for which appropriate treatment could not be planned due to insufficient time to perform essential examinations to establish anal cancer staging. These include high-resolution magnetic resonance imaging to assess the tumor’s location and its locoregional anatomical relationships and contrast-enhanced computed tomography to evaluate distant metastatic disease^44^.

The survival probability of cases that underwent surgery was similar to those treated with chemoradiotherapy. However, it is important to note that the 95%CI for the estimate of cases treated with surgery was wider, which consequently increases its margin of imprecision. Therefore, it is reasonable to conjecture that these were cases with tumors smaller than 1 cm , which were treated this way because, in such situations, the 5-year survival rate is similar to that provided by chemoradiotherapy (83.5 vs. 86.8%, respectively)^7^.

The 5-year overall survival observed in this study is similar to that reported in national^11^ and international studies^2,27,28^, and in some cases, even higher^12,24^. It is noteworthy that, despite being conducted at a High-Complexity Oncology Care Center within the SUS, the study reports survival rates comparable to those seen in high-income countries like the United States^2,27^ and England^28^, a country that, like Brazil, has a universal and free healthcare system.

Female cases with advanced staging showed a higher probability of survival and almost a 50% lower risk of death compared to males. Hospital- and population-based studies conducted in the United States^2,19^, Australia^18,40^, France^3^, and Norway^14^ corroborate these findings, regardless of staging. Therefore, it is reasonable to speculate that the reason for the sex difference in anal cancer survival is that women tend to pay more attention to their own health, seeking and accessing healthcare services more frequently than men^9^. Even so, further research is necessary to better understand this phenomenon. In this context, it is worth mentioning the recommendation of the Brazilian Society of Surgical Oncology that female anal cancer cases undergo gynecological examination with screening for cervical, vulvar, and vaginal cancers^44^, due to the association between anal cancer and human papillomavirus (HPV) infection, which is primarily transmitted through sexual contact^34,44^.

Squamous cell carcinoma is the most common histological type of anal cancer^13,23,33,36,44^, with more than 90% of cases associated with HPV, especially HPV-16, a high-risk oncogenic viral type^25,38,34^. Although there is evidence of increasing incidence^10,39^, anal cancer is relatively uncommon in the general population, being more frequent among men who have sex with men, people living with the human immunodeficiency virus (HIV), immunosuppressed patients (including solid organ and bone marrow transplant recipients), and women with a history of neoplastic or pre-neoplastic vulvar lesions^8^. This scenario underscores the importance of adopting anal cancer screening strategies for these high-risk population groups^21,44^.

Anal cancer is a disease whose most common symptoms (pain, bleeding, and a sensation of a rectal/anal mass) are associated with other less severe conditions, which can lead to delayed diagnosis, often at a locally advanced stage^44^. Thus, the best way to prevent more people from suffering its effects is through primary prevention: HPV vaccination and the use of condoms during sexual intercourse. In Brazil, the vaccine against the most common types of HPV (6, 11, 16, and 18) is provided free of charge by the SUS — just like male and female condoms^4^ — and is recommended for girls and boys aged 9–14 years, as well as people living with HIV, solid organ/bone marrow transplant recipients, cancer patients, and sexual abuse victims aged 9–45 years^5^.

The results of the present study should be interpreted considering its limitations and strengths. The decision to conduct stratified analyses by staging was prompted by its main limitation: the absence of information on this variable in nearly half of the sample. In an attempt to reduce the number of cases with unknown staging, efforts to match TNM classification with staging were made but failed due to the significant lack of TNM data in the database provided by the Hospital-Based Cancer Registry. On the contrary, including cases with unknown staging in the stratified Cox models was a strength, as it minimized potential lead-time bias^17^, did not reduce the power of the analyses by decreasing the sample size^22^, and accounted for the statistical difference observed in the variable treatment received between staging groups (known vs. unknown; Table 2).

Another point to note is that the survival analysis was not disease-specific, as it was not possible to identify the cause of death for all cases in the cohort. Additionally, cases censored due to loss of active follow-up, despite having been followed for mean and median times exceeding 3 years, may have underestimated the survival probability and introduced bias due to differential follow-up losses^6^.

Another limitation concerns the use of secondary data. In this study, data systematically collected from patient records by the Hospital-Based Cancer Registry are primarily used to monitor the quality of care provided^15^, which made it impossible to evaluate the influence of important variables associated with anal cancer survival, such as tumor size and HPV-16 infection^33^. Despite the unavailability of other relevant variables for a more satisfactory outcome analysis — a limitation of any retrospective cohort study^26^ — the results of this study have expanded knowledge about hospital survival of anal cancer in Brazil by analyzing data from a large sample of cases treated by the SUS at a reference center for cancer treatment located in one of Brazil’s largest states, thus qualifying it as the most significant study on this topic ever conducted in the country.

Finally, the improvement of record-keeping in patient files—especially concerning the clinical staging of câncer—should be continuously promoted among healthcare professionals working in High-Complexity Oncology Care Centers. In addition to being frequently used for research purposes, this document is the primary data source for the Hospital-Based Cancer Registry.

## CONCLUSIONS

The findings of this study support the superiority of chemoradiotherapy over other therapeutic modalities for anal cancer, resulting in greater survival and a better prognosis.
